# Urban Wild Boars and Risk for Zoonotic *Streptococcus suis*, Spain

**DOI:** 10.3201/eid2406.171271

**Published:** 2018-06

**Authors:** Xavier Fernández-Aguilar, Marcelo Gottschalk, Virginia Aragon, Jordi Càmara, Carmen Ardanuy, Roser Velarde, Nuria Galofré-Milà, Raquel Castillo-Contreras, Jorge R. López-Olvera, Gregorio Mentaberre, Andreu Colom-Cadena, Santiago Lavín, Oscar Cabezón

**Affiliations:** Centre de Recerca en Sanitat Animal (CReSA, IRTA-UAB), Bellaterra, Spain (X. Fernández-Aguilar, V. Aragon, N. Galofré-Milà, O. Cabezón);; Universitat Autònoma de Barcelona, Bellaterra (X. Fernández-Aguilar, R. Velarde, R. Castillo-Contreras, J.R. López-Olvera, G. Mentaberre, A. Colom-Cadena, S. Lavín, O. Cabezón);; Université de Montréal, St.-Hyacinthe, Québec, Canada (M. Gottschalk);; Hospital Universitari de Bellvitge, L’Hospitalet de Llobregat, Spain (J. Càmara, C. Ardanuy);; CIBER de Enfermedades Respiratorias, Madrid, Spain (C. Ardanuy)

**Keywords:** bacterial meningitis, disease transmission, zoonoses, native invader, public health, Spain, Streptococcus suis, Sus scrofa, wild boars, urban wildlife, wildlife conflict, bacteria, meningitis/encephalitis, streptococci

## Abstract

Urban wild boars (*Sus scrofa*) from Barcelona, Spain, harbor great diversity of *Streptococcus suis* strains, including strains with the *cps*2 gene and with the same molecular profile as local human cases. The increasing trend of potential effective contacts for *S. suis* transmission is of public health concern.

Populations of the European wild boar (*Sus scrofa*) have been increasing (*1*). The wild boar’s high adaptability to human-dominated landscapes and its increase in human tolerance have also prompted its presence in urban areas, leading to conflicts with local humans (*2*,*3*). Health risk assessment should therefore be performed in this new scenario, in which wild boars and their interactions with humans are becoming common in heavily populated areas (*3*).

*Streptococcus suis* is an emerging zoonotic pathogen (*4*). Among the bacterium’s 35 described serotypes, serotypes 2, 5, and 14 are those most related to human disease (*4*,*5*). Several risk factors have been associated with *S. suis* infection in humans, such as consumption of undercooked pig meat or work in pig-related occupations (*5*). The wild boar has also been identified as a source of human infection in relation to hunting activities (*6*). However, the increasing presence of synanthropic wild boars may also pose a novel public health risk for nonhunters. The objective of this study was to investigate the presence of *S. suis* strains with zoonotic potential in wild boars from the metropolitan area of Barcelona, Spain, and assess the risk of transmission to humans.

## The Study

**Figure 1:**
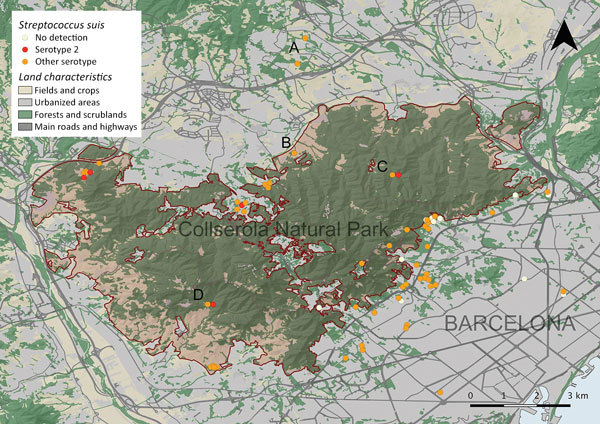
Part of the metropolitan area of Barcelona, Spain, showing land characteristics, Collserola Natural Park, the location of the wild boars sampled, and results of *Streptococcus suis* serotype 2 strains, identified by both isolation and molecular detection. Letters indicate locations where several wild boars were sampled, obtained by box traps (A, n = 21) or regular hunting campaigns (B, n = 9; C, n = 14; D, n = 5).

The metropolitan area of Barcelona is heavily populated (>3.2 million) and includes Collserola Natural Park, 8,000 hectares of Mediterranean forest (Figure 1). The wild boar population in this forest is ≈1,200, and habituation to humans has become common (*2*). A total of 108 apparently healthy wild boars were sampled in the Barcelona area for the presence of *S. suis* during April–November 2015; the animals had been hunted in regular campaigns, captured by box traps and euthanized for population control, or captured with the aid of anesthetic darts and euthanized because of public risk. All procedures were performed under the regulation of the competent public administrations and in compliance with current guidelines for ethical use of animals in research, following European (2010/63/EU) and Spanish (R.D. 53/2013) legislation. 

At postmortem examination, we collected tonsil and nasal swab specimens from each wild boar and cultured them on chocolate agar. For each sample, a maximum of 4 presumptive *S. suis* colonies were subcultured and subsequently identified with a *S. suis*–specific gene *recN* PCR (*7*). We molecularly serotyped *Streptococcus suis* isolates (*8*) and further serotyped positive isolates for *cps*2 gene by coagglutination (*9*). We also included 2 local *S. suis* serotype 2 strains of human origin for molecular characterization (Table 1). The source of the infection was not identified in 1 of these 2 patients (*10*), but the second patient acquired the infection through an accidental cut on his hand with a tusk while manipulating a wild boar hunted 50 km from Barcelona. In the days immediately following his injury, this patient developed pain in his hand; 5 days after the injury, he sought care at an emergency department. He had fever (body temperature 39.5°C), leukocytosis (35 × 10^9^ cells/L; reference range 3.9–10 × 10^9^ cells/L), limited arm mobility, headache, and lethargic mental status. At this stage, the patient received diagnoses of arthritis and meningitis.

**Table 1:** Sequence type and virulence associated gene profile of the *Streptococcus suis* serotype 2 strains isolated from wild boars and humans in metropolitan area of Barcelona, Spain, 2012–2015*

Source	Year	Clinical infection	MLST	Virulence genes			References
*mrp*	*ef*	*sly*
Human male, 57 y of age	2012	Meningitis, arthritis, bacteremia	ST3	+	+	+	(*10*)
Human male, 48 y of age	2014	Meningitis, arthritis	ST1	+	+	+	This study
Wild boar	2015	No	ST1	+	+	+	This study
Wild boar	2015	No	ST1	+	+	+	This study
Wild boar	2015	No	ST1	+	+	+	This study

*MLST, multilocus sequence typing; ST, sequence type.

We isolated *S. suis* from 91 (84.3%; 95% CI 76.2%–89.9%) wild boars, identifying 332 isolates from tonsil (n = 141) and nasal (n = 191) swab specimens. We found 17 different serotypes and nontypeable strains in the wild boar population (Table 2). Three of the colonies were serotype 2, isolated from 2 wild boars, and were confirmed along with the human strains as serotype 2 by coagglutination test. We further characterized serotype 2 strains (both wild boar and human origin) using multilocus sequence typing (*11*) and by assessing the presence of the genes muramidase-released protein (*mrp*), extracellular factor (*ef*), and hemolysin suilysin (*sly*), associated with invasiveness and zoonotic potential (*12*). These serotype 2 strains belonged to clonal complex 1 and had the same molecular characterization (sequence type [ST] 1; *cps*2/*mrp*+/*ef*+/*sly*+), with the exception of 1 human isolate that was ST3 (a single-locus variant of ST1) (Table 1). Results from enterobacterial repetitive intergenic consensus (ERIC) PCR (*13*) showed a unique fingerprint profile for all serotype 2 strains and 32 different fingerprints for 36 other isolates (originating from 10 wild boars). In addition, 4 of 94 wild boars (4.3%; 95% CI 1.7%–10.4%) tested positive for detection of the *cps2* gene directly in nasal swab specimens.

**Table 2:** Frequency of *Streptococcus suis* serotypes identified by multiplex PCR in isolates from 108 wild boars from the metropolitan area of Barcelona, Spain, 2012–2015*

Serotype	Isolates					Wild boar	
No. nasal samples positive	No. tonsillar samples positive	Total no. samples positive	Prevalence, % (95% CI)	No. animals positive*	Prevalence % (95% CI)
2	2	1	3	0.9 (0.3–2.6)		2	1.8 (0.5–6.5)
4	3	7	10	3.0 (1.6–5.4)		10	9.2 (5.1–16.2)
6	5	0	5	1.5 (0.6–3.5)		2	1.8 (0.5–6.5)
7	0	2	2	0.6 (0.2–2.2)		2	1.8 (0.5–6.5)
8	3	1	4	1.2 (0.5–3.1)		3	2.8 (0.9–7.8)
9	7	18	25	7.5 (5.1–10.9)		13	12.0 (7.2–19.5)
10	4	1	5	1.5 (0.6–3.5)		3	2.8 (0.9–7.8)
12	0	1	1	0.3 (0.0–1.7)		1	0.9 (0.0–5.1)
15	0	4	4	1.2 (0.5–3.1)		3	2.8 (0.9–7.8)
16	27	7	34	10.2 (7.4–14.0)		17	15.7 (10.1–23.8)
17	1	0	1	0.3 (0.0–1.7)		1	0.9 (0.0–5.1)
21	30	0	30	9.0 (6.4–12.6)		12	11.1 (6.5–18.4)
23	3	0	3	0.9 (0.3–2.6)		3	2.8 (0.9–7.8)
27	2	2	4	1.2 (0.5–3.1)		4	3.7 (1.4–9.1)
28	1	2	3	0.9 (0.3–2.6)		3	2.8 (0.9–7.8)
31	20	13	33	9.9 (7.1–13.6)		23	21.3 (14.6–29.9)
33	3	0	3	0.9 (0.3–2.6)		2	1.8 (0.5–6.5)
Nontypeable	80	82	162	48.8 (43.5–54.1)		70	64.8 (55.4–73.2)
Total	191	141	332	100		108†	100

*Number of wild boars that were positive for that serotype, either from nasal or tonsillar isolates. †Total count does not coincide with the column sum because each wild boar can harbor >1 serotype and *S. suis* was not isolated from 17 wild boars.

**Figure 2:**
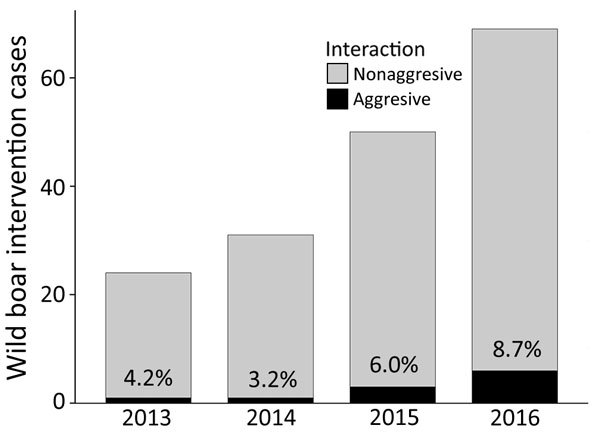
Occurrence and temporal trend of veterinary interventions related to wild boar removal in Barcelona, Spain, 2013–2016, and percentage of interventions with aggressive interactions. Aggressive interaction involves both violent physical contacts (charging or pushing for food) and aggressions to humans (bites).

To evaluate the occurrence and trend of potential effective contacts between urban wild boars and humans, we examined 174 veterinary field interventions related to wild boar incidents in Barcelona during 2013–2016. We found that both the number of interventions and the percentage of interventions motivated by violent physical contacts or aggressive interactions with humans (charges, bites, or injuries) showed an increasing trend (Figure 2).

## Discussion

The isolation of *S. suis* in apparently healthy wild boars from the metropolitan area of Barcelona highlights its reservoir role and identifies this species as a potential source for human infections in urban areas. The high number of wild boars carrying *S. suis* confirms results obtained in a previous study, in which *S. suis* and *S. suis*–like strains were detected together through the use of a PCR for the *gdh* gene (*14*). Our results show a high diversity of *S. suis* serotypes, with and without pathogenic relevance for humans (*4*). A high percentage of isolates was nontypeable, as previously found in both domestic pig and wild boar carriers (*4*). These results further suggest that *S. suis* and serotype 2 strains are widely distributed in European wild boar populations (*14*).

Most human disease cases reported worldwide are associated with invasive serotype 2 strains that typically cause meningitis and, ultimately, neurologic sequelae or death (*4*,*5*). ST1, belonging to clonal complex 1, is one of the most frequent sequence types found in human and pig disease cases from Europe and other regions; it often involves the encoding virulence genes *mrp*, *ef*, and *sly* found in *cps2*-positive wild boar strains (*15*). Despite the high diversity of genomic fingerprints found in wild boar isolates, a unique fingerprint included all serotype 2 strains, suggesting no consistent differences between human and wild boar isolates. However, wild boars harboring *cps2*-positive strains were apparently healthy carriers. Other serotypes found in apparently healthy wild boars, such as 5, 9, 14, 16, and 21, have also been occasionally associated with severe human disease (*4*,*5*).

At least 7 human cases of wild boar bites were treated at the emergency department of a main local hospital (2012–2015, Communication Unit of Vall d’Hebron, Barcelona), yet small wounds not attended in health centers can be enough to acquire systemic infections (*4*). Human *S. suis* infections are associated mainly with pig farming and the food industry, being considered an occupational disease or foodborne disease in developing countries (*5*). The increasing trend of potential effective contacts for *S. suis* transmission between urban wild boars and humans in Barcelona suggests that *S. suis* can be acquired by humans who belong to apparently low-risk collectives.

## Conclusions

Wild boars from the metropolitan area of Barcelona harbor *S. suis* strains with the same enterobacterial repetitive intergenic consensus PCR fingerprints and virulence gene profiles as invasive strains of local human origin; thus, they have zoonotic and invasive potential. The increasing interactions of wild boars with humans in urban areas pose risks for *S. suis* or other directly or indirectly transmitted zoonoses, making the presence of urban wild boars a public health concern.
